# Compression therapy using surgical gloves does not prevent paclitaxel-induced peripheral neuropathy: results from a double-blind phase 2 trial

**DOI:** 10.1186/s12885-021-08240-6

**Published:** 2021-05-13

**Authors:** Haruru Kotani, Mitsuo Terada, Makiko Mori, Nanae Horisawa, Kayoko Sugino, Ayumi Kataoka, Yayoi Adachi, Naomi Gondou, Akiyo Yoshimura, Masaya Hattori, Masataka Sawaki, Chihoko Takahata, Makiko Kobara, Hiroji Iwata

**Affiliations:** 1 Department of Breast Oncology, Aichi Cancer Center Hospital, 1-1 Kanokoden, Chikusa-ku, Nagoya, 464-8681 Japan; 2 Department of Breast Surgery, Nagoya City University Graduate School of Medicine, 1, Kawasumi, Mizuho-cho, Mizuho-ku, Nagoya, 467-8601 Japan; 3 Department of Outpatient Treatment Center, Aichi Cancer Center Hospital, 1-1 Kanokoden, Chikusa-ku, Nagoya, 464-8681 Japan; 4 Nursing Department, Aichi Cancer Center Hospital, 1-1 Kanokoden, Chikusa-ku, Nagoya, 464-8681 Japan

**Keywords:** Breast cancer, Paclitaxel, Prevention, Chemotherapy-induced peripheral neuropathy, Surgical gloves

## Abstract

**Background:**

Chemotherapy-induced peripheral neuropathy (CIPN) is a common adverse effect of paclitaxel (PTX). There is no known prophylactic measure, although there are some reports of prevention with compression therapy using surgical gloves. On account of its predominantly subjective symptoms, it is difficult to exclude bias when assessing for CIPN. In this study, we assessed the effectiveness of the same procedure for the prevention of paclitaxel-induced PN based on a double-blind study design.

**Methods:**

The patients with early and recurrent breast cancer (with no prior PTX exposure) initiating weekly chemotherapy with PTX 80 mg/m^2^ were enrolled. Each patient donned two gloves on each hand at every PTX infusion. Two one-size-smaller gloves were donned on one hand (study side) and two normal-size gloves were donned on the other hand (control side) during 90 min from 30 min before the infusion to 30 min after the end of the infusion. Study side are blind for both patients and assessing physicians according to determination of the study side by research nurses in the chemotherapy unit.

The primary outcome was the difference in the frequency of CIPN (motor/sensory) determined by the physician using the common terminology criteria for adverse events **(**CTCAE v4.0), with an evaluation at each cycle of PTX infusion. McNemar test was used to assess the primary outcome.

**Results:**

Between July 2017 and November 2018, 56 patients were enrolled and 49 patients were evaluated. Overall, Grade ≥ 2 PN (sensory) was observed in 30.6 and 36.7% in the study and control sides, respectively (McNemar *p* = 0.25). PN (motor) was observed in 4.1 and 6.1% in the study and control sides, respectively (McNemar *p* = 1.0).

**Conclusion:**

Surgical glove compression therapy showed no statistically significant effect on the incidence of PTX-induced PN.

**Trial registrations:**

This study was registered with the University Hospital Medical Information Network (UMIN) Clinical Trials Registry managed by the National University Hospital Council of Japan (UMIN000027944). Registered 26 June 2017.

## Background

Breast cancer is the most common cancer among women worldwide. As the survival rates are increasing, improving the patient’s quality of life (QOL) became more important.

Chemotherapy-induced peripheral neuropathy (CIPN) is one of the most important adverse event effects on QOL of breast cancer patients [1]. Although paclitaxel (PTX) is one of the key drugs for primary and metastatic breast cancer [2, 3],it is well known that PTX often causes peripheral neuropathy (PN) [4]. Despite several reports about the prevention of the CIPN, the management of that remains an unsolved problem [5–7]. The current American Society of Clinical Oncology Clinical Guidelines for CIPN management recommend duloxetine for treatment of moderate CIPN; however, there are no recommended agents for its prevention [8].

Recently, the effectiveness of compression therapy for nanoparticle albumin-bound-paclitaxel- (nab-PTX) induced PN using surgical gloves was reported in Japan [9]. In that study, compression therapy was performed as follows: subjects donned two one-size-smaller surgical gloves on their dominant hand and left the other hand bare as a control. They confirmed that this compression therapy using gloves significantly reduced the incidence of grade ≥ 2 common terminology criteria for adverse events (CTCAE) PN from 76.1 to 21.4% for nanoparticle albumin-bound-paclitaxel (nab-PTX). Although compression therapy is a low-cost and safe method, we consider the accurate assessment of CIPN to be difficult because it is evaluated according to subjective symptoms; therefore, some biases, such as performance bias and detection bias, could occur [10].

In this study, we assessed the effectiveness of compression therapy using gloves (same procedure as previous reports) for the prevention of CIPN based on complete blinding for patients and the assessing physician under a study design which aimed to minimize these biases.

## Methods

This trial was done at Aichi Cancer Center Hospital in Japan. The study population consisted of previously PTX-naïve adults (aged ≥20 y) with early and/or recurrent/advanced breast cancer starting chemotherapy with weekly PTX (80 mg/m^2^). One cycle of chemotherapy was either one dose every 3 weeks or administration on days 1, 8, and 15 every 4 weeks. Patients with human epidermal growth factor receptor 2 (HER2)-positive breast cancer received trastuzumab (initial dose, 8 mg/kg; loading dose, 6 mg/kg every 3 weeks) with/without pertuzumab (initial dose, 840 mg; loading dose, 420 mg every 3 weeks) and recurrent/advanced breast cancer patients received bevacizumab (10 mg/kg, day 1 and day 15 every 4 weeks) subsequent to the administration of paclitaxel [11].

Inclusion criteria were Eastern Cooperative Oncology Group (ECOG) performance status of 0–1, and adequate bone marrow, hepatic, renal, and heart function. Exclusion criteria were prior treatment with PTX or nab-PTX, grade ≥ 1 peripheral neuropathy according to CTCAE caused by any reason, difficulty securing vascular access outside of the hand, and allergy to surgical gloves.

### Procedure

The procedure was a modification of a previous study assessing the effect of surgical gloves on one hand for nab-PTX [9]. In our study, each patient donned two gloves on each hand at every PTX infusion. Two one-size-smaller gloves were donned on one hand (study side) and two normal-size gloves were donned on the other hand (control side) over the 90 min from 30 min before the infusion to 30 min after the end of the infusion. The study side and control sides were blinded to both patients and physicians by research nurses at the chemotherapy unit. Patients were randomly assigned to right hand or left hand tight glove groups (one-to-one ratio). Further, to minimize dominant hand bias, research nurses divided the groups such that 50% of subjects had the tighter gloves on the dominant hand and 50% had the tighter gloves on the nondominant hand. The size of gloves was determined by research nurses after the patients tried to fit them several times. They ensured that same side to be study side each time using correspondence table which was blinded for the patients. As the source of the gloves, Emblem surgical gloves (Sanko Chemical Industrial, Hiroshima, Japan) was used for 5.0 size, Techwrap® F4 (Hogy Medical, Tokyo, Japan) was used for gloves of 5.5 size and above.

Following the previous study, we also evaluated the temperature at the tip of each finger. Images of the palmar aspects of both hands were taken by a thermographic camera (FLIR C2, FLIR Systems, Inc. Oregon, USA) before donning and just before removal of the gloves. The temperature at the end of each infusion was measured with gloves donned. The point of measurement was between the distal interphalangeal joint and the base of the nail. Any differences between the study side and control side fingertip temperature changes were assessed.

### Statistical analysis

The primary outcome was the difference in the incidence of Grade ≥ 2 CIPN (motor/sensory) between the study side and control side as determined by the physician using CTCAE v4.0. Secondary outcome was the difference in the incidence of CIPN (motor/sensory) between the study side and control side as assessed by the patient using the Patient-reported Outcomes version of CTCAE (PRO-CTCAE) [12, 13]. For severity assessment, ≥ “moderate,” and for interference with daily activities assessment, ≥ “somewhat” was considered as an event.

Assessment was done at the time of the first day of each cycle and after the end of the four PTX courses (before the next treatment administration). Since we focused on prevention, the study was ended when the subject developed a primary outcome in either of their hands. All patients who could complete the first cycle of the study drug were assessed for the primary outcome.

We planned samples by assessing the frequency of grade ≥ 2 PN in the control hand as 30% based on previous reports [3, 14, 15]. According to the previous studies of compression therapy, we expected a 15% difference between each side with 80% power and a significance level for a two-sided test of 0.05. We assumed a 5% dropout rate, and planned a total sample size of 55 patients.

McNemar’s chi-squared test was used to assess the primary and secondary outcomes. Paired *t*-tests were used to assess fingertip temperature change.

All statistical analyses were carried out using STATA ver.12 (StataCorp, College Station, TX, USA). All tests were two-sided and *p* < 0.05 were considered statistically significant.

The protocol was approved by the institutional review boards of the Aichi Cancer Center Hospital. All patients provided written informed consent.

## Results

### Characteristics of patients and controls

Between July 11, 2017 and November 26, 2018, 56 patients were enrolled in the study. Two patients who complained of discomfort donning the gloves and four patients who could not complete the first cycle of PTX due to liver enzyme elevation, allergy to PTX, or intolerance to alcohol (two patients) were excluded. As a result, 49 patients were evaluated. Regarding patient characteristics (Table 1), median age was 53 years, 43 patients (87.8%) were early and 6 patients (12.2%) were recurrent/advanced breast cancer patients. A total of 21 patients underwent axillary dissection. There was no need for dosage reduction of PTX during the observation period.

**Table 1:** Patient characteristics

Median age	52.5 (23–74)	Treatment	
Menoposal status		Neoadjuvant chemotherapy	11 (22.5%)
premenopausal	21 (42.9%)	Adjuvant chemotherapy	32 (65.3%)
postmenopausal	28 (57.1%)	Recurrence chemotherapy	6 (12.2%)
ECOG performance		Combined therapy	
0	48 (98.0%)	Trastuzumab	27 (55.1%)
1	1 (2.0%)	Bevasizumab	6 (12.2%)
Dominant hand		No combined therapy	16 (32.7%)
R	48 (98.0%)	History of chemotherapy	
L	1 (2.0%)	No	20 (40.8%)
Glove size(control side)		Yes	29 (59.2%)
5.5	17 (73.9%)	Previous chemotehrapy regimen	
6.0	23 (46.9%)	Anthracyclin based	28 (57.1%)
6.5	9 (18.4%)	Docetaxel	1 (2.0%)
Location of primary tumor		Smoking status	
R	18 (36.7%)	Never smoker	36 (73.5%)
L	30 (61.2%)	Current smoker	3 (6.1%)
Bilateral	1 (2.0%)	Former smoker	6 (12.2%)
Axillary dissection		Unknown	4 (8.2%)
No	26 (55.3%)	Diabetes	
Yes	21 (44.7%)	No	45 (91.8%)
Subtype of the primary tumor		Yes	4 (8.2%)
ER+/HER2-	11 (22.9%)		
ER+/HER2+	18 (37.5%)		
ER−/HER2+	8 (16.7%)		
ER−/HER2-	11 (22.9%)		

### Primary outcome

**Fig. 1:**
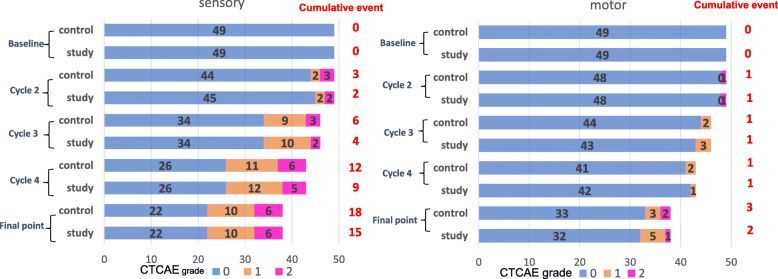
Primary outcome: difference in the frequency of CIPN (motor/sensory). At the final assessed point, Grade ≥ 2 or more PN (sensory) was observed in 30.6 and 36.7% patients on the study and control side, respectively (McNemar *p* = 0.25). PN (motor) was observed in 4.1 and 6.1% on the study and control side, respectively (McNemar *p* = 1.0). No statistically significant difference in primary endpoints was observed between the control side and the study side

Overall, 18 patients (36.7%) developed Grade ≥ 2 sensory PN in their control hand and 15 patients (30.6%) developed the event in their study hand (McNemar *p* = 0.25) (Fig. 1). Of these patients, 15 developed the event on both sides at the same time and 3 developed the event only on the control side.

For motor PN, three patients (6.1%) developed Grade ≥ 2 motor PN in their control side hand and two patients (4.1%) developed the event in their study side hand. (McNemar *p* = 1.0). Of these patients, two developed the event on both sides at the same time and one developed the event only on the control side. No statistically significant difference in primary outcome was observed between the control side and the study side.

### Secondary outcome

**Fig. 2:**
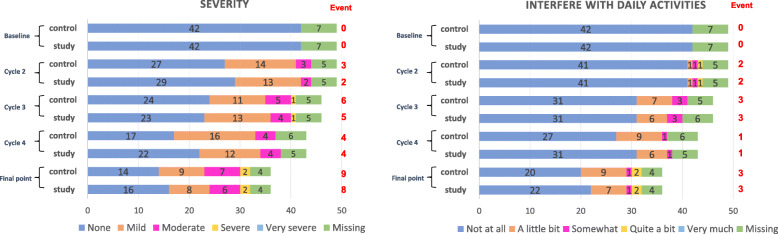
Secondary outcome: difference in the frequency of CIPN assessed using PRO-CTCAE. The questionnaire completion rate was 85.2%. There was no difference between the study and control sides in either the severity of numbness (McNemar *p* = 1.0) or interference with daily activities. (McNemar *p* = 1.0)

For the Pro-CTCAE assessment, the questionnaire completion rate was 85.2%. As shown in Fig. 2, there was also no difference between the study and control side in either the grade of numbness (McNemar *p* = 1.0) or the effect on activities of daily living (McNemar, *p* = 1.0).

### Temperature changes

**Fig. 3:**
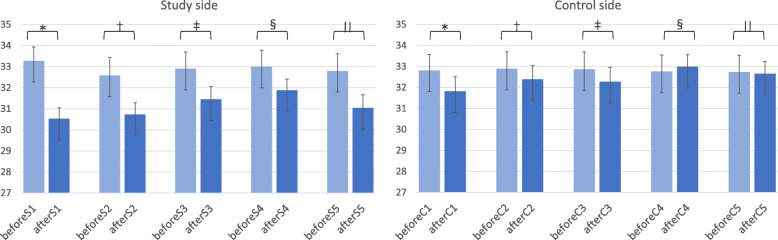
Temperature changes in each fingertip. For the study side, the temperature of all fingertips was decreased (1.1–2.7 °C). For the control side, the temperature change was relatively small (− 0.23 − + 0.99 °C). *p* = 0.0004 (* first digit), 0.0508 († second digit), 0.1226 (‡ third digit), 0.0136 (§fourth digit), and0.0020 (|| fifth digit)

To confirm the reproducibility of the compression therapy procedure with the previous study, we measured temperature changes on each fingertip in 24 patients (Fig. 3). For the study side, the temperature of all fingertips was decreased (− 1.1– − 2.7 °C) after the administration of PTX. For the control side, the temperature change was relatively small (− 0.23 − + 0.99 °C). The changes in temperature were significant in the first (* *p* = 0.0004), fourth (§ *p* = 0.0136), and fifth (|| *p* = 0.0020) digits.

## Discussion

In this study, we found that SG compression therapy was not effective in preventing the incidence of PTX-induced PN. The results of temperature changes on each fingertip were consistent with the previous report, confirming the reproducibility of the compression therapy method using gloves.

The most difficult problem when addressing CIPN is that it is largely subjective [16, 17]. In this study, to reduce differences in primary outcome evaluation between physicians, we confirmed the interpretation of CTCAE v4 before starting the trial as follows: since grade 1 is described as “asymptomatic,” ≥ Grade 2 could be applicable when the patient developed any definite subjective symptoms. To confirm the accuracy of the results, we also observed the consistency between the primary endpoint (objective evaluation) and secondary endpoint (subjective evaluation).

Due to the difficulty of standardizing the objective measure, various biases could occur in clinical trials assessing CIPN [8]. The strength of our study is that it succeeded in minimizing bias by using a randomized control trial design.

Our trial had several limitations. First, there was the possibility that donning correctly-sized gloves for the control side may also be effective in preventing CIPN. However, the incidence of CIPN on the control side did not deviate substantially from that of previous reports [3, 14], and we observed a definite difference in fingertip temperatures between the study and control sides. Further, although this study was blinded to the patients, they might infer the study side because of its greater tightness. If such inference existed, the effect did not seem to affect the negative results due to the performance bias considered to work to increase the difference of results between study side and control side. Second, there was a slight difference in the method of measuring the temperature compare to the previous reports. In previous reports, the temperature at the end of the chemotherapy infusion was measured with cutting the top of the SG of each finger by scissors. However, we could not do the same procedure because it was dangerous to cut the fingertips of tightened fit gloves. We observed that there was no difference between bared and SG donned fingertip temperature before starting this trial. Third, although the study and control side were blinded for the assessing physician, we could not confirm inter-rater agreement due to lack of the second assessing physician. Forth, we could not deny all the biases. For example, the performance bias on both sides could not be denied.

The difference between our study and previous studies warrant mention. First, the chemotherapy agent was different. Tsuyuki et al. confirmed the effectiveness of compression therapy in preventing CIPN with nab-PTX; in their study, the incidence of grade ≥ 2 PN ranged from 45 to 76%, which is higher than the incidence with PTX [18–21]. The inconsistency in the results may be due to this difference in CIPN incidence between nab-PTX and PTX. Second, the studies differed in design, albeit that the method of compression therapy was same. Following the first report, which confirmed effectiveness by using gloves on the dominant hand only, they also confirmed the effectiveness by wearing gloves on both hands [22]. Neither study designs allowed the exclusion of performance bias and detection bias due to the lack of blinding of patients and physicians. Third, since we focused on prevention, patients who developed Grade 2 PN were removed from the study. In contrast, in a previous study, the procedure was continued after the development of PN, and the authors observed a lower incidence of each grade. Fourth, the studies seemed to differ with regard to the measurement of glove size. In our study, 34.7% of patients required a size 5.0 glove for the study side, although no patient in the previous studies required this size [9, 22]. Since all three studies were performed in Japan, we believe that there was no substantial difference in patients’ physiques. Although our study revealed the ineffectiveness of gloves for preventing CIPN of PTX, the measurement of glove size might be a problem to be addressed. Additionally, there were two patients who complained of discomfort while donning and preferred to leave the trial, while there was no patient who was unable to tolerate donning the gloves in the previous two studies.

## Conclusions

In conclusion, compression therapy using tight gloves was not effective for decreasing the incidence of PTX-induced PN. We confirmed the reproducibility of the method of compression therapy using thermography. Further studies are needed to elucidate the prevention of CIPN from PTX.

## Data Availability

The datasets used and/or analysed during the current study are available from corresponding author on reasonable request.

## Abbreviations

CIPN: Chemotherapy-induced peripheral neuropathy
CTCAE: common terminology criteria for adverse events
ECOG: Eastern Cooperative Oncology Group
HER2: human epidermal growth factor receptor 2
PN: peripheral neuropathy
PTX: paclitaxel

## References

[1] Rivera DR, Ganz PA, Weyrich MS, Bandos H, Melnikow J. Chemotherapy-associated peripheral neuropathy in patients with early-stage breast Cancer: a systematic review. J Natl Cancer Inst. 2018;110(2). 10.1093/jnci/djx140. 10.1093/jnci/djx140 PMC5825681 28954296

[2] Mamounas EP, Bryant J, Lembersky B, Fehrenbacher L, Sedlacek SM, Fisher B, Wickerham DL, Yothers G, Soran A, Wolmark N (2005). Paclitaxel after doxorubicin plus cyclophosphamide as adjuvant chemotherapy for node-positive breast cancer: results from NSABP B-28. J Clin Oncol.

[3] Sparano JA, Wang M, Martino S, Jones V, Perez EA, Saphner T, Wolff AC, Sledge GW, Wood WC, Davidson NE (2008). Weekly paclitaxel in the adjuvant treatment of breast cancer. N Engl J Med.

[4] Tanabe Y, Hashimoto K, Shimizu C, Hirakawa A, Harano K, Yunokawa M, Yonemori K, Katsumata N, Tamura K, Ando M, Kinoshita T, Fujiwara Y (2013). Paclitaxel-induced peripheral neuropathy in patients receiving adjuvant chemotherapy for breast cancer. Int J Clin Oncol.

[5] Sundar R, Bandla A, Tan SS, Liao LD, Kumarakulasinghe NB, Jeyasekharan AD, Ow SG, Ho J, Tan DS, Lim JS, Vijayan J, Therimadasamy AK, Hairom Z, Ang E, Ang S, Thakor NV, Lee SC, Wilder-Smith EP (2016). Limb hypothermia for preventing paclitaxel-induced peripheral neuropathy in breast Cancer patients: a pilot study. Front Oncol.

[6] Kuriyama A, Endo K (2018). Goshajinkigan for prevention of chemotherapy-induced peripheral neuropathy: a systematic review and meta-analysis. Support Care Cancer.

[7] Ibrahim EY, Ehrlich BE (2020). Prevention of chemotherapy-induced peripheral neuropathy: a review of recent findings. Crit Rev Oncol Hematol.

[8] Hershman DL, Lacchetti C, Dworkin RH, Lavoie Smith EM, Bleeker J, Cavaletti G, Chauhan C, Gavin P, Lavino A, Lustberg MB, Paice J, Schneider B, Smith ML, Smith T, Terstriep S, Wagner-Johnston N, Bak K, Loprinzi CL, American Society of Clinical O (2014). Prevention and management of chemotherapy-induced peripheral neuropathy in survivors of adult cancers: American Society of Clinical Oncology clinical practice guideline. J Clin Oncol.

[9] Tsuyuki S, Senda N, Kanng Y, Yamaguchi A, Yoshibayashi H, Kikawa Y, Katakami N, Kato H, Hashimoto T, Okuno T, Yamauchi A, Inamoto T (2016). Evaluation of the effect of compression therapy using surgical gloves on nanoparticle albumin-bound paclitaxel-induced peripheral neuropathy: a phase II multicenter study by the Kamigata breast Cancer study group. Breast Cancer Res Treat.

[10] Agabegi SS, Stern PJ (2008). Bias in research. Am J Orthop (Belle Mead NJ).

[11] Miller K, Wang M, Gralow J, Dickler M, Cobleigh M, Perez EA, Shenkier T, Cella D, Davidson NE (2007). Paclitaxel plus bevacizumab versus paclitaxel alone for metastatic breast cancer. N Engl J Med.

[12] Miyaji T, Iioka Y, Kuroda Y, Yamamoto D, Iwase S, Goto Y, Tsuboi M, Odagiri H, Tsubota Y, Kawaguchi T, Sakata N, Basch E, Yamaguchi T (2017). Japanese translation and linguistic validation of the US National Cancer Institute's patient-reported outcomes version of the common terminology criteria for adverse events (PRO-CTCAE). J Patient Rep Outcomes.

[13] Shimozuma K, Ohashi Y, Takeuchi A, Aranishi T, Morita S, Kuroi K, Ohsumi S, Makino H, Mukai H, Katsumata N, Sunada Y, Watanabe T, Hausheer FH (2009). Feasibility and validity of the patient neurotoxicity questionnaire during taxane chemotherapy in a phase III randomized trial in patients with breast cancer: N-SAS BC 02. Support Care Cancer.

[14] Seidman AD, Berry D, Cirrincione C, Harris L, Muss H, Marcom PK, Gipson G, Burstein H, Lake D, Shapiro CL, Ungaro P, Norton L, Winer E, Hudis C (2008). Randomized phase III trial of weekly compared with every-3-weeks paclitaxel for metastatic breast cancer, with trastuzumab for all HER-2 overexpressors and random assignment to trastuzumab or not in HER-2 nonoverexpressors: final results of Cancer and leukemia group B protocol 9840. J Clin Oncol.

[15] Lee JJ, Swain SM (2006). Peripheral neuropathy induced by microtubule-stabilizing agents. J Clin Oncol.

[16] Alberti P, Rossi E, Cornblath DR, Merkies IS, Postma TJ, Frigeni B, Bruna J, Velasco R, Argyriou AA, Kalofonos HP, Psimaras D, Ricard D, Pace A, Galie E, Briani C, Dalla Torre C, Faber CG, Lalisang RI, Boogerd W, Brandsma D, Koeppen S, Hense J, Storey D, Kerrigan S, Schenone A, Fabbri S, Valsecchi MG, Cavaletti G, Group CI-P (2014). Physician-assessed and patient-reported outcome measures in chemotherapy-induced sensory peripheral neurotoxicity: two sides of the same coin. Ann Oncol.

[17] Flatters SJL, Dougherty PM, Colvin LA (2017). Clinical and preclinical perspectives on chemotherapy-induced peripheral neuropathy (CIPN): a narrative review. Br J Anaesth.

[18] Gradishar WJ, Tjulandin S, Davidson N, Shaw H, Desai N, Bhar P, Hawkins M, O'Shaughnessy J (2005). Phase III trial of nanoparticle albumin-bound paclitaxel compared with polyethylated castor oil-based paclitaxel in women with breast cancer. J Clin Oncol.

[19] Rivera E, Cianfrocca M (2015). Overview of neuropathy associated with taxanes for the treatment of metastatic breast cancer. Cancer Chemother Pharmacol.

[20] Gradishar WJ, Krasnojon D, Cheporov S, Makhson AN, Manikhas GM, Clawson A, Bhar P, McGuire JR, Iglesias J (2012). Phase II trial of nab-paclitaxel compared with docetaxel as first-line chemotherapy in patients with metastatic breast cancer: final analysis of overall survival. Clin Breast Cancer.

[21] Nakamura S, Iwata H, Funato Y, Ito K, Ito Y (2015). Results of a drug use investigation of nanoparticle albumin-bound paclitaxel for breast cancer. Gan To Kagaku Ryoho.

[22] Tsuyuki S, Yamagami K, Yoshibayashi H, Sugie T, Mizuno Y, Tanaka S, Kato H, Okuno T, Ogura N, Yamashiro H, Takuwa H, Kikawa Y, Hashimoto T, Kato T, Takahara S, Katayama T, Yamauchi A, Inamoto T (2019). Effectiveness and safety of surgical glove compression therapy as a prophylactic method against nanoparticle albumin-bound-paclitaxel-induced peripheral neuropathy. Breast.

